# Barriers to Clinical Trial Participation: Comparative Study Between Rural and Urban Participants

**DOI:** 10.2196/33240

**Published:** 2022-04-21

**Authors:** Dinesh Pal Mudaranthakam, Byron Gajewski, Hope Krebill, James Coulter, Michelle Springer, Elizabeth Calhoun, Dorothy Hughes, Matthew Mayo, Gary Doolittle

**Affiliations:** 1 University of Kansas Medical Center Kansas City, KS United States

**Keywords:** rural residents, clinical trials, screening, cancer, patients, lung cancer, health policy epidemiology, cancer patients, electronic screening logs, electronic screening

## Abstract

**Background:**

The National Clinical Trials Network program conducts phase 2 or phase 3 treatment trials across all National Cancer Institute’s designated cancer centers. Participant accrual across these clinical trials is a critical factor in deciding their success. Cancer centers that cater to rural populations, such as The University of Kansas Cancer Center, have an additional responsibility to ensure rural residents have access and are well represented across these studies.

**Objective:**

There are scant data available regarding the factors that act as barriers to the accrual of rural residents in these clinical trials. This study aims to use electronic screening logs that were used to gather patient data at several participating sites in The Kansas University of Cancer Center’s Catchment area.

**Methods:**

Screening log data were used to assess what clinical trial participation barriers are faced by these patients. Additionally, the differences in clinical trial participation barriers were compared between rural and urban participating sites.

**Results:**

Analysis revealed that the hospital location rural urban category, defined as whether the hospital was in an urban or rural setting, had a medium effect on enrolment of patients in breast cancer and lung cancer trials (Cohen *d*=0.7). Additionally, the hospital location category had a medium effect on the proportion of recurrent lung cancer cases at the time of screening (*d*=0.6).

**Conclusions:**

In consideration of the financially hostile nature of cancer treatment as well as geographical and transportation barriers, clinical trials extended to rural communities are uniquely positioned to alleviate the burden of nonmedical costs in trial participation. However, these options can be far less feasible for patients in rural settings. Since the number of patients with cancer who are eligible for a clinical trial is already limited by the stringent eligibility criteria required of such a complex disease, improving accessibility for rural patients should be a greater focus in health policy.

## Introduction

There are numerous barriers for rural residents to obtain health care. Some of the barriers include but are not limited to lack of facilities, lack of infrastructure, inability to travel, lack of specialists, financial barriers, and limited access to clinical trials [1]. Consequently, patients may avoid or delay care, resulting in more severe clinical outcomes [2,3].

Within this field, there are several environmental risk factors such as sun exposure, pesticide exposure, and risk of injury from farming equipment [4,5]. Among these risks, pesticides and other chemicals may lead to an increased cancer incidence among rural populations [6]. Given the nature of cancer, without early diagnosis, the patient might be left with fewer treatment options or may even run out of treatment options. Moreover, treatments for battling cancer are very expensive as they require multiple sessions over a long period of time [7,8]. The medications involved with cancer treatment are also expensive, and not all are covered through medical insurance leaving the patient to pay for it [9]. Given most of the rural residents are either self-employed or employed through small companies, typically their insurance coverage is very minimal [10]. A lack of insurance coverage or gaps in insurance coverage can add to the difficulty of the treatment process for rural patients. In many cases, these patients must choose between skipping treatment or taking on debt [9]. In consideration of these obstacles, clinical trials may represent an underutilized avenue of affordable treatment for rural patients. However, the availability of these trials to rural patients is limited by the logistic difficulty of bringing expensive medical devices involved in cancer treatment to isolated health centers in nonmetro areas.

The Masonic Cancer Alliance (MCA), which serves as the outreach network for the University of Kansas Cancer Center (KUCC), already has a great relationship with most of the rural hospitals and clinics in the catchment area. The KUCC launched this network to extend clinical trials at these hospitals and clinics in rural and health professional shortage areas. The majority of trials made available to the MCA sites are the National Cancer Institute’s National Clinical Trials Network studies. To better understand the volume and patient cohort availability, all of the screening information gathered at these locations was documented at each of the sites under a screening log database. These community sites span across the state of Kansas, covering the majority of KUCC’s catchment area.

The National Clinical Trials Network (NCTN) program is aimed to motivate like-minded people across North America and internationally to coordinate and support cancer clinical trials that are funded by the National Cancer Institute. The trials that were part of the NCTN program were used as potential trials available for patients who received care at 9 community sites. The community site information is summarized in Table 1, including the county and state these sites are located, as well as their Rural Urban Continuum Codes (RUCC) classification, which designates counties as rural or urban depending on population and urbanization.

**Figure 1:**
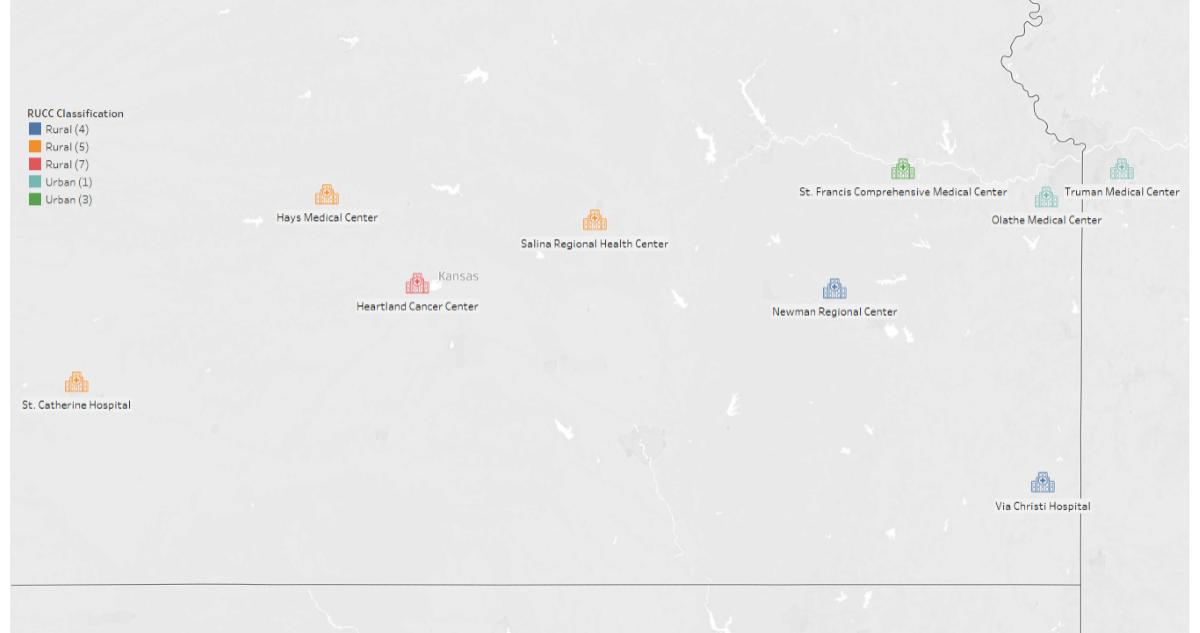
Geographical location of the clinical trial screening sites. RUCC: Rural Urban Continuum Codes. ©Mapbox ©OpenStreetMap.

KUCC, in collaboration with MCA, launched clinical trial screening at the 9 community sites that are located across the KUCC catchment area for the NCTN trials. Figure 1 provides a geographical representation of where these sites are located.

**Table 1:** Community partner sites where participants were screened

Site name	County, state (population)	RUCC^a^ classification	Health professional shortage areas (primary care)
Hays Medical Center	Ellis County, KS (28,553)	Rural (5)	No
Heartland Cancer Center	Finney County, KS (36,467)	Rural (5)	Yes
Newman Regional Center	Lyon County, KS (33,195)	Rural (4)	Yes
Olathe Medical Center	Johnson County, KS (602,401)	Urban (1)	No
Salina Regional Health Center	Saline County, KS (54,224)	Rural (5)	Yes
St. Catherine Hospital	Finney County, KS (36,467)	Rural (5)	Yes
St. Francis Comprehensive Medical Center	Shawnee County, KS (176,875)	Urban (3)	Yes
Truman Medical Center	Jackson County, MO (703,011)	Urban (1)	No
Via Christi Hospital	Crawford County, KS (38,818)	Rural (4)	Yes

^a^RUCC: Rural Urban Continuum Codes.

## Methods

### Screening Methodology

The MCA, in conjunction with the Biostatistics and Informatics Shared Resources, have built a screening log survey using REDCap (Research Electronic Data Capture) [11]. The screening log was targeted to capture high-level information about participants who were screened at these community sites. The screening log captured information such as whether there was a trial available based on the community cancer center’s clinical trials portfolio. If a potential trial was currently available for a participant’s cancer type, the participants were screened and screening information was documented. Documented screening information for these patients included cancer disease type, stage, and recurrence. The screening log is attached as a supplementary document listing all the questions that were captured during the screening. If a patient was found to be ineligible for a trial after screen, the corresponding reasons were also documented. If a patient was eligible for a trial but chose not to take part, their reasons were also documented. Multiple disease trials were considered to be available trials for both patients with lung cancer and patients with breast cancer.

**Figure 2:**
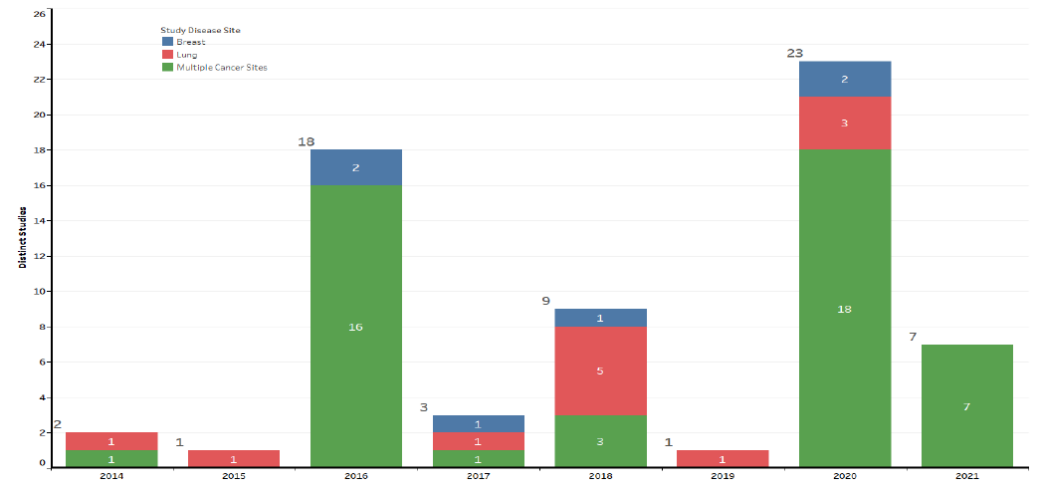
Clinical trials actively screening during the calendar year.

The University of Kansas Medical Center Institutional Review Board’s approval was given to capture the participants screening information across the 9 community sites in October 2014. Since then, information has been captured under the REDCap screening log project. The data dictionary depicting the screening information that was captured has been attached as a supplementary document (Multimedia Appendix 1). The number of clinical trials that were available across these 9 sites is illustrated as a bar chart in Figure 2. These results are stratified by year, and different colors represent the type of disease the trial was targeting (breast, lung, or multiple disease). The multiple disease trials are broader studies that allowed screening for both breast and lung but also other common cancer types.

The 9 sites involved in the screening process span across the state of Kansas and are described in Table 1. Based on the RUCC, these sites were classified as Rural (RUCC 4-9) or Urban sites (RUCC 1-3). For the purposes of this study, we used hospital location to categorize rural or urban status to compare factors in breast cancer and lung cancer between the rural and urban groups. These factors include clinical trial availability, barriers to treatment, and disease characteristics.

### Statistical Analysis

The data capture for screening were developed with a pure intention of operational goals, and consequently there was not a formal study design to determine the sample size for each of these sites. Moreover, the screening process of clinical trials is hard to predict, and there is always an ebb and flow with screening both in urban and rural areas. Due to these sampling issues, the Fisher exact test *P* value was determined to be an insufficient method for comparing rural participants to urban participants. Additionally, in consideration of the fact that significant *P* values are also likely to be found in large sample sizes even when the size of the effect is negligible, Cohen *d* was used to calculate effect size instead [12]. To obtain the Cohen *d*, a log odds ratio was calculated and then converted [13]. A Cohen *d* value of [0;0.2) implies negligible effect; [0.2; 0.5) implies small effect; [0.5; 0.8) implies medium effect; and [0.8; infinity) implies large effect [14].

### Cohen d Calculation

Cohen *d* is calculated using the following standard formula:



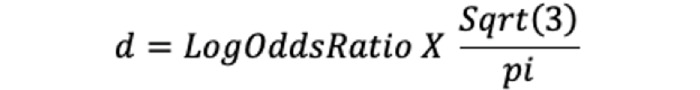





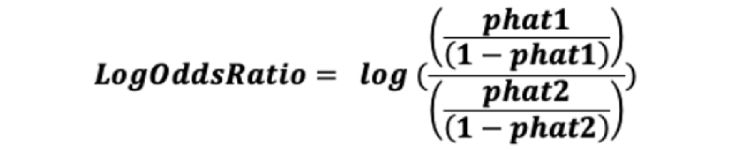



Variables included for analysis included the rural-urban category, with outcomes including the disease-specific information gathered during the screening process. Among the disease-specific information, variables varied between patients who had breast cancer and those diagnosed with lung cancer.

Outcome variables for patients with breast cancer included clinical trial availability, whether they were a new or existing patient at diagnosis, tumor stage, histology of the breast, nodal breast status, metastatic status, recurrence status, stage of breast, and hormone of the breast. Clinical trial availability was recorded as yes or no depending on whether a clinical trial was available. Metastatic status was recorded as yes or no. Recurrence status was recorded as recurrent or nonrecurrent. Tumor stage was recorded as T1, T2, T3, or T4. Histology of the breast was recorded as ductal carcinoma in situ (invasive carcinoma), or inflammatory carcinoma. Nodal breast status was recorded as either positive or negative. Stage of breast was recorded as 0, I, II, III, or IV. Lastly, the hormone of the breast was recorded as ER/PR+ (estrogen receptor/progesterone receptor) HER2+ (human epidermal growth factor receptor 2), ER/PR+ HER2–, ER/PR– HER2+, or ER/PR– HER2–.

Outcome variables for patients with lung cancer included clinical trial availability, whether they were a new or existing patient at diagnosis, tumor stage, histology of the lung, nodal lung status, metastatic status, and recurrence status. Clinical trial availability was recorded as yes or no depending on whether a clinical trial was available. Metastatic status was recorded as yes or no. Recurrence status was recorded as recurrent or nonrecurrent. Lung histology was recorded as adenocarcinoma, bronchoalveolar, squamous cell carcinoma, small-cell carcinoma, or mesothelioma. Tumor stage was recorded as T0, T1, T2, T3, or T4. Lastly, nodal lung status was recorded either positive or negative.

### Ethics Approval

The University of Kansas Medical Center granted approval under a central IRB with reliance by the other institutions (STUDY00002341).

## Results

A total of 2258 patients with breast cancer and 1347 patients diagnosed with lung cancer were screened across 9 sites from October of 2014 to December of 2020. Some common reasons why patients were not able to participate in clinical trials are described in Multimedia Appendix 2. As stated previously, we sought to assess the relative availability of clinical trials between rural and urban patients. Additionally, we analyzed the relative incidence of certain cancer disease features between these two populations. These results are detailed in Multimedia Appendix 3.

Among patients with breast cancer, we noted significant differences in clinical trial availability between rural-urban categories. For urban residents, 177 (18.7%) of the 945 patients with breast cancer were eligible for a clinical trial based on their portfolio. Compare this to rural residents, where 79 (6.01%) of 1313 patients were eligible for a clinical trial. A Cohen *d* value of 0.7 represents a medium effect between the rural and urban groups when it comes to clinical trial availability. Using the Cohen *d* calculation formula, this would mean that an urban patient who has breast cancer would be 3.56 more likely to have an available clinical trial for their cancer type compared to a rural patient with breast cancer. This suggests that an urban participant diagnosed with breast cancer had higher odds of finding a potential clinical trial compared to a rural patient diagnosed with the same condition. Hospital Location Rural-Urban Category (HLRUC) had a small effect on whether a patient was a new or existing patient at the time of diagnosis (Cohen *d*=0.2), suggesting slightly higher odds that a rural patient would be a new patient at the time of diagnosis. Health risk control did not display an effect on either the stage of breast cancer or breast histology. For both outcomes, the Cohen *d* was 0.1. Health risk control displayed a small effect size (Cohen *d*=0.2-0.4) on nodal breast status, metastatic status, recurrence status, stage of breast, and hormone of breast. This suggests slightly higher odds for the incidence of these outcomes among rural patients diagnosed with breast cancer.

Among patients with lung cancer, there was a similar disparity in clinical trial availability. For rural patients with lung cancer, 84 (10.5%) of 798 patients had an available clinical trial. For urban patients with lung cancer, 140 (43%) of 325 patients had an available clinical trial. The residence category resulted in a Cohen *d* of 0.8, which would mean that urban patients with lung cancer were 4.268 times more likely to have an available clinical trial. HLRUC had a small effect on the incidence of lung histology categories including adenocarcinoma, bronchoalveolar, small-cell carcinoma, and mesothelioma. HLRUC did not influence the lung histology category of squamous cell carcinoma. HLRUC had a small effect on incidence of the T1 stage of lung cancer (Cohen *d*=0.2) but had no effect on the incidence of other stages. HLRUC had no effect on nodal status (Cohen *d*=0.1), and a small effect on metastatic status (Cohen *d*=0.2). HLRUC had a medium effect on recurrent status of patients with lung cancer (Cohen *d*=0.6), suggesting a higher odds of recurrent lung cancer among rural patients.

## Discussion

### Key Findings

Our results suggest that clinical trial availability was greater for urban patients with breast cancer and lung cancer than it was for their rural counterparts. It stands to reason that the benefit of expanding clinical trial availability to rural patients could be significant for an already underserved population. Since the screening was a part of the data gathering process, the effect size could also potentially be due to fewer study options that are available at the rural sites. Stringent eligibility criteria are a long-standing barrier in cancer trial participation, and there have been recent initiatives to reevaluate and broaden clinical trial availability [15,16]. Broadening the criteria has multiple benefits such as improved clinical trial participation, reflecting larger patient population and increasing patient access to new investigational treatment [17]. Even after initial prescreening, the participants might have to undergo a set of labs before they are officially enrolled into the clinical trial. Costs for these additional labs or exams might not be covered by the clinical trial sponsor and might discourage participants from even entertaining the idea of participation into these trials [18]. Subsequent studies should consider barriers to clinical trial participation in the context of cancer stage as well as current factors. In cases where the participants’ diagnosis is in an advanced stage, they have very fewer clinical trial opportunities because of fewer advanced stage trials and the aggressive nature of the disease [19]. The time-sensitive nature of advanced stage cancer incentivizes physicians to begin treatment as quickly as possible instead of searching for potential clinical trials. When there are additional barriers complicating clinical trial participation, this could make clinical trials particularly unavailable for patients in an advanced cancer stage.

Apart from the clinical trial availability metric, our keen focus was to assess if patients who seek care in rural areas might differ in care, which could potentially lead to malignancy of cancer or a diagnosis of a late stage. Our analysis indicated that the prevalence of certain cancer features was similar between populations seeking care at rural and urban centers. However, the limited sample size of patients at rural locations could affect the interpretation of these results. More data from rural populations, as well as the inclusion of additional factors in the screening process, will be required for future analysis.

Recent studies suggest that involving primary care physicians in the conversation of clinical trial participation can encourage rural patients to see cancer trials as a treatment option [20]. For rural participants who are diagnosed with cancer for the first time, they may lack the experience and information to decide what treatment options suit them. This can exacerbate the already present barriers to clinical trial participation for these patients. If information on clinical trial options is provided to them by a primary care physician or other familiar health care worker, they may be more receptive to alternate treatment options such as clinical trial participation [21]. In this way, some of the individual and personal barriers to clinical trial participation can be alleviated.

Multimedia Appendix 2 illustrates some of the common reasons why participants were not able to find an appropriate clinical trial that suits their profile. Additionally, if they were qualified for study participation and decided not to participate, those reasons have been documented as well. Among both the breast and the lung cancer group, the major screening failure reason has been the performance status or the ECOG (Eastern Cooperative Oncology Group) status. The ECOG status is a frequently used measure in clinical trial planning, which details a patient’s ability to care for themselves, as well as their mobility and activity levels. Typically, most trials under their inclusion criteria look for participants who have a lower performance status; a higher performance status would mean they are limited self-care or need additional support [22].

As mentioned previously, multiple disease trials were considered to be available trials for patients with lung cancer and those with breast cancer. While the lack of specificity in these trials allows for greater accessibility, the broadness of their typical premise means the potential benefits of participation are limited.

Some of the common reasons why the patient decided not to participate includes “time concern,” “travel concern,” “insurance denial,” “study logistics,” “language barrier,” “social,” and “physician didn’t offer.” One of the low hanging fruits that can be easily addressed from the above barriers is to educate the physicians at these sites and provide them with a comprehensive list of studies that suits their patient’s profile. For this very reason, KUCC has developed a mobile app also known as “Clinical Trial Finder App” that can be used by any physician to easily screen or refer a patient while the patient is in the clinic with them [23,24].

### Limitations

Due to the data limitation, we are unable to assess if the screening rate varies by site or based on race or ethnicity. As a future project, our team proposes to find ways to collaborate with these sites to gain additional demographics and clinical information to dive deeper into understanding the various trends. Another major limitation of the study was that hospital location was used as a surrogate for patient residence. In future studies, it would be beneficial to gather data on actual patient residence in order to determine urban or rural residence categories. The screening estimates might be on the lower end, as some of the screened patients who did not follow the standard screening procedures could have been excluded from the data capture system.

### Conclusion

Even in this day and age, we continue to observe barriers that discourage participants from participating in clinical trials. Additionally, the health care availability gap between rural and urban participants is widening, which limits the generalizability of clinical trials for rural participants. Technological, therapeutic, and medical practice advances have had very little impact on reducing these barriers. A few of the notable barriers include lack of personnel to screen participants, lack of technology, commuting issues, and differences among the population characteristics. We as a cancer center strive to continue educating our clinical teams at the rural sites about the potential referral opportunities. Future policy makers must consider more targeted programs that facilitate the participation of rural patients. This approach must be multifaceted, involving earning the trust of rural patients, providing resource to facilitate clinical trial participation, disseminating the right information, and continuing to engage and adapt to the dynamic rural environment. Additional support must be provided to encourage clinical trial participation through resources such as transportation, childcare, and tax credits, among others.

## Appendix

Multimedia Appendix 1 Survey template.

Multimedia Appendix 2 Screen failure reason and qualified participants reason to decline trial participation.

Multimedia Appendix 3 Comparison of rural versus urban participants based on the participants' screening characteristics.

## Abbreviations

ECOG: Eastern Cooperative Oncology Group
ER/PR: estrogen receptor/progesterone receptor
HER2: human epidermal growth factor receptor 2
HLRUC: Hospital Location Rural-Urban Category
KUCC: University of Kansas Cancer Center
MCA: Masonic Cancer Alliance
NCTN: National Clinical Trials Network
REDCap: Research Electronic Data Capture
RUCC: Rural Urban Continuum Codes
