# Supplementary material for: Barriers to Clinical Trial Participation: Comparative Study Between Rural and Urban Participants
Source: JMIR Cancer. 2022 Apr 21;8(2):e33240. doi: 10.2196/33240 (PMC9073606; doi:10.2196/33240)
Supplement: Multimedia Appendix 1 [file cancer_v8i2e33240_app1.pdf]

Confidential

MCA Clinical Trials Screening Log

Record ID	__________________
Patient Initials	__________________
Patient Status	◯ New Patient<br>◯ Established Patient
Cancer Site	◯ Brain<br>◯ Breast<br>◯ GI<br>◯ GU<br>◯ GYN<br>◯ Head and Neck<br>◯ Heme<br>◯ Lung<br>◯ Melanoma / Sarcoma
Comments	______________________
BRAIN	
Histology	◯ Glioblastoma (WHO Grade IV)<br>◯ Oligodendroglioma<br>◯ Astrocytoma<br>◯ Anaplastic Glioma (WHO Grade III)<br>◯ Low Grade Glioma (WHO Grade II)<br>◯ Meningioma<br>◯ Other
Other_brain_histology	__________________
Stage	◯ New diagnosis<br>◯ Recurrent / progression
BREAST	
Histology	◯ DCIS<br>◯ Invasive Carcinoma<br>◯ Inflammatory Carcinoma
Tumor Stage	◯ T1<br>◯ T2<br>◯ T3<br>◯ T4
Nodal status	◯ Negative<br>◯ Positive

Metastatic	◯ M0<br>◯ M1
Recurrent / Progression	◯ Yes<br>◯ No
Stage	◯ 0<br>◯ I<br>◯ II<br>◯ III<br>◯ IV
Hormone Recepter Status	◯ ER/PR + HER2 +<br>◯ ER/PR + HER2 -<br>◯ ER/PR - HER2 +<br>◯ ER/PR - HER2 - (triple neg)
LUNG	
Histology	◯ Adenocarcinoma<br>◯ Bronchoalveolar<br>◯ Squamous cell carcinoma<br>◯ Small cell carcinoma<br>◯ Mesothelimoa
Tumor Stage	◯ T1<br>◯ T2<br>◯ T3<br>◯ T4
Nodal status	◯ N0<br>◯ N1<br>◯ N2<br>◯ N3
Metastatic	◯ M0<br>◯ M1
Recurrent / Progression	◯ Yes<br>◯ No
Genomic / Marker Status	◯ ALK +<br>◯ ALK -<br>◯ EGFR +<br>◯ EGFR -<br>◯ KRAS+<br>◯ KRAS-<br>◯ CEA elevated
GU	
Location	◯ Renal (kidney)<br>◯ Bladder / Ureteral<br>◯ Prostate<br>◯ Testes<br>◯ Penis<br>◯ Urethra

Histology	◯ Clear cell<br>◯ Papillary<br>◯ Transitional cell<br>◯ Small cell<br>◯ Neuroendocrine<br>◯ Adenocarcinoma<br>◯ Squamous cell<br>◯ Seminomatous<br>◯ Nonseminomatous
Tumor Stage	◯ T1<br>◯ T2<br>◯ T3<br>◯ T4
Nodal status	◯ N0<br>◯ N1<br>◯ N2<br>◯ N3
Metastatic	◯ M0<br>◯ M1
Recurrent / Progression	◯ Yes<br>◯ No
Genomic / Marker Status	◯ PSA elevated
GI	
Location	◯ Esophagus<br>◯ Stomach<br>◯ Liver<br>◯ Gallbladder<br>◯ Pancreas<br>◯ Peritoneum<br>◯ Small Intestine<br>◯ Colon<br>◯ Rectal<br>◯ Anus<br>◯ Other GI
Tumor Stage	◯ T1<br>◯ T2<br>◯ T3<br>◯ T4
Nodal status	◯ N0<br>◯ N1<br>◯ N2<br>◯ N3
Metastatic	◯ M0<br>◯ M1
Recurrent / Progression	◯ Yes<br>◯ No

Stage	◯ I<br>◯ II<br>◯ III<br>◯ IV
Genomic / Marker Status	◯ KRAS +<br>◯ KRAS -
GYN	
Location	◯ Ovary<br>◯ Fallopian tubes<br>◯ Uterine cervix<br>◯ Uterine other<br>◯ Vagina<br>◯ Vulva<br>◯ Primary Peritoneal
Histology	◯ Squamous<br>◯ Adenocarcinoma<br>◯ Clear cell<br>◯ Serous<br>◯ Mucinous<br>◯ Endometroid<br>◯ Transitional cell<br>◯ Mesenchymal<br>◯ Mixed
Tumor Stage	◯ T1<br>◯ T2<br>◯ T3<br>◯ T4
Nodal Status	◯ N0<br>◯ N1<br>◯ N2<br>◯ N3
Metastatic	◯ M0<br>◯ M1
Recurrent / Progression	◯ Yes<br>◯ No
Stage	◯ I<br>◯ II<br>◯ III<br>◯ IV
Genomic / Marker Status	◯ CA-125 elevated
HEAD & NECK	
Location	◯ Oral cavity & pharynx<br>◯ Larynx<br>◯ Thyroid<br>◯ Nasal/Sinus<br>◯ Other

Histology	◯ Squamous<br>◯ Adenocarcinoma<br>◯ Transitional cell<br>◯ Spindle cell<br>◯ Keratinizing<br>◯ Undifferentiated<br>◯ Mixed
Tumor Stage	◯ T1<br>◯ T2<br>◯ T3<br>◯ T4
Nodal Status	◯ N0<br>◯ N1<br>◯ N2<br>◯ N3
Metastatic	◯ M0<br>◯ M1
Recurrent / Progression	◯ Yes<br>◯ No
Stage	◯ I<br>◯ II<br>◯ III<br>◯ IV
HEME	
Location	◯ Lymphoma<br>◯ Leukemia<br>◯ Myeloma
Histology	◯ DLCBL<br>◯ Follicular<br>◯ Hodgkins<br>◯ Mantle Cell
Stage	◯ I<br>◯ II<br>◯ III<br>◯ IV
Recurrent / Progression	◯ Yes<br>◯ No
Histology	◯ CLL<br>◯ CML<br>◯ AML<br>◯ ALL<br>◯ Other
Treatment Status	◯ Untreated<br>◯ Recurrent / Progression

Histology	◯ Myeloma<br>◯ Amyloidosis<br>◯ MGUS
Treatment Status	◯ Smoldering / Observation<br>◯ Untreated<br>◯ Recurrent / Progression
MELANOMA / SARCOMA	
Histology	◯ Melanoma<br>◯ Osteoblastic sarcoma<br>◯ Fibroblastic sarcoma<br>◯ Chondroblastic sarcoma<br>◯ Ewings sarcoma
Stage	◯ Low grade stage I<br>◯ High grade stage II<br>◯ Metastatic stage III
Stage	◯ I<br>◯ II<br>◯ III<br>◯ IV
Recurrent / Progression	◯ Yes<br>◯ No
Nodal Status	◯ Negative<br>◯ Postive
Genomic / Marker Status	◯ BRAF -<br>◯ BRAF +
Clinic Visit Date	______________________<br>(M-D-Y of first clinic visit for new pt)
Clinical Trial Available	◯ Yes<br>◯ No<br>((based on above criteria))
Clinical Trial Name or Number Being considered	______________________

If patient did not meet trial eligibility, please select all that apply	☐ Age<br>☐ Performance Status<br>☐ Abnormal labs<br>☐ Abnormal organ function (cardiac, renal, lung, etc.)<br>☐ Pregnant/Nursing<br>☐ Other Cancer/Previous Cancer<br>☐ Uncontrolled Metastases (CNS)<br>☐ Previous Chemotherapy<br>☐ Previous Radiotherapy<br>☐ Prior Surgery<br>☐ Psychiatric Condition<br>☐ Will be Treated at another Clinic/Facility<br>☐ Inadequate tissue available (refused re-biopsy or further tissue collection)<br>☐ Other (specify)
Signed Consent	○ Yes<br>○ No
Did participant enroll on study	○ Yes<br>○ No
Reason not consented	☐ No Insurance<br>☐ Insurance Denial<br>☐ Time Concern<br>☐ Travel Concern<br>☐ Fear of Side Effects<br>☐ Unsure of Treatment/Clinical Trials in General<br>☐ Didn't Want to Risk Placebo<br>☐ Wanted a Different Treatment<br>☐ Family didn't want them to participate<br>☐ Study Logistics (e.g. drug ordering delay, baseline scans out of timeframe)<br>☐ Social Issues (housing, childcare)<br>☐ Language barrier<br>☐ Physician Did Not Offer (specify in notes)<br>☐ Did not meet Trial Eligibility
Physician did not offer	______________________________
Other reasons not consented	______________________________
