# Supplementary material for: Barriers to Clinical Trial Participation: Comparative Study Between Rural and Urban Participants
Source: JMIR Cancer. 2022 Apr 21;8(2):e33240. doi: 10.2196/33240 (PMC9073606; doi:10.2196/33240)
Supplement: Multimedia Appendix 2 [file cancer_v8i2e33240_app2.docx]

| **Screen Failure Reason (Breast)** | **Counts** |  | **Screen Failure Reason (Lung)** | **Counts** |
| --- | --- | --- | --- | --- |
| Age | 6 |  | Age | 8 |
| Performance Status | 19 |  | Performance Status | 71 |
| Abnormal labs | 11 |  | Abnormal labs | 4 |
| Abnormal organ function (cardiac, renal, lung, etc.) | 11 |  | Abnormal organ function (cardiac, renal, lung, etc.) | 15 |
| Pregnant/Nursing | 0 |  | Pregnant/Nursing | 0 |
| Other Cancer/Previous Cancer | 10 |  | Other Cancer/Previous Cancer | 13 |
| Uncontrolled Metastases (CNS) | 5 |  | Uncontrolled Metastases (CNS) | 7 |
| Previous Chemotherapy | 21 |  | Previous Chemotherapy | 3 |
| Previous Radiotherapy | 3 |  | Previous Radiotherapy | 3 |
| Prior Surgery | 5 |  | Prior Surgery | 0 |
| Psychiatric Condition | 1 |  | Psychiatric Condition | 5 |
| Will be Treated at another Clinic/Facility | 13 |  | Will be Treated at another Clinic/Facility | 8 |
| Inadequate tissue available (refused re-biopsy or further tissue collection) | 0 |  | Inadequate tissue available (refused re-biopsy or further tissue collection) | 7 |
| Cancer not correct stage/type | 29 |  | Cancer not correct stage/type | 14 |
| Patient declined treatment | 9 |  | Patient declined treatment | 7 |
| Different treatment/trial | 7 |  | Different treatment/trial | 8 |
| Logistical Issue | 10 |  | Logistical Issue | 3 |
| No Comment | 8 |  | No Comment | 3 |
| Medicine Conflict | 2 |  | Medicine Conflict | 1 |
| Margins Issue | 1 |  | Margins Issue | 2 |
| Finished Treatment | 2 |  | Finished Treatment | 1 |
| Physician Recommended against | 1 |  | Physician Recommended against | 1 |
| No Surgical Option | 1 |  | No Surgical Option | 1 |
| Fear of Placebo | 2 |  | Fear of Placebo | 0 |
| Insurance Issue | 1 |  | Insurance Issue | 0 |
| **Reason Not Consented (Breast)** | **Counts** |  | **Reason Not Consented (Lung)** | **Counts** |
| No Insurance | 0 |  | No Insurance | 0 |
| Insurance Denial | 0 |  | Insurance Denial | 1 |
| Time Concern | 5 |  | Time Concern | 0 |
| Travel Concern | 1 |  | Travel Concern | 1 |
| Fear of Side Effects | 8 |  | Fear of Side Effects | 2 |
| Unsure of Treatment/Clinical Trials in General | 10 |  | Unsure of Treatment/Clinical Trials in General | 8 |
| Didn't Want to Risk Placebo | 3 |  | Didn't Want to Risk Placebo | 0 |
| Wanted a Different Treatment | 15 |  | Wanted a Different Treatment | 9 |
| Family didn't want them to participate | 2 |  | Family didn't want them to participate | 0 |
| Study Logistics (e.g. drug ordering delay, baseline scans out of timeframe) | 8 |  | Study Logistics (e.g. drug ordering delay, baseline scans out of timeframe) | 3 |
| Social Issues (housing, childcare) | 2 |  | Social Issues (housing, childcare) | 2 |
| Language barrier | 2 |  | Language barrier | 0 |
| Physician Did Not Offer (specify in notes) | 13 |  | Physician Did Not Offer (specify in notes) | 13 |
| Did not meet Trial Eligibility | 127 |  | Did not meet Trial Eligibility | 115 |
