# Supplementary material for: Barriers to Clinical Trial Participation: Comparative Study Between Rural and Urban Participants
Source: JMIR Cancer. 2022 Apr 21;8(2):e33240. doi: 10.2196/33240 (PMC9073606; doi:10.2196/33240)
Supplement: Multimedia Appendix 3 [file cancer_v8i2e33240_app3.docx]

| **Cancer** | **Variable** | **Variables** | **Rural** | **Urban** | **Total** | **Fisher’s p-value** | **Absolute Cohens d** | **Cohens d** | **Lower 95% Confidence Interval** | **Upper 95% Confidence Interval** |
| --- | --- | --- | --- | --- | --- | --- | --- | --- | --- | --- |
| ***Breast*** |  |  |  |  |  |  |  |  |  |  |
|  | clinical_trial_available |  |  |  |  | <0.05 |  |  |  |  |
|  |  | Yes | 79 | 177 | 256 |  |  |  |  |  |
|  |  |  | 6% | 19% |  |  |  |  |  |  |
|  |  | No | 1234 | 768 | 2002 |  | 0.706 | 0.706 | 0.552 | 0.861 |
|  |  |  | 94% | 81% |  |  |  |  |  |  |
|  | patient_status |  |  |  |  | <0.05 |  |  |  |  |
|  |  | New Patient | 1064 | 893 | 1957 |  |  |  |  |  |
|  |  |  | 82% | 90% |  |  |  |  |  |  |
|  |  | Established Patient | 227 | 101 | 328 |  | 0.350 | 0.350 | 0.212 | 0.488 |
|  |  |  | 18% | 10% |  |  |  |  |  |  |
|  | histology_breast |  |  |  |  | <0.05 |  |  |  |  |
|  |  | Ductal carcinoma in situ | 210 | 141 | 351 |  | 0.115 | 0.115 | -0.407 | 0.638 |
|  |  |  | 16% | 14% |  |  |  |  |  |  |
|  |  | Invasive Carcinoma | 1066 | 875 | 1941 |  | 0.080 | -0.080 | -0.603 | 0.442 |
|  |  |  | 83% | 85% |  |  |  |  |  |  |
|  |  | Inflammatory Carcinoma | 7 | 13 | 20 |  |  |  |  |  |
|  |  |  | 1% | 1% |  |  |  |  |  |  |
|  | tumor_stage_breast |  |  |  |  | 0.115 |  |  |  |  |
|  |  | Tumor 1 | 647 | 497 | 1144 |  | 0.095 | -0.095 | -0.461 | 0.272 |
|  |  |  | 58% | 63% |  |  |  |  |  |  |
|  |  | Tumor 2 | 353 | 225 | 578 |  | 0.094 | 0.094 | -0.272 | 0.460 |
|  |  |  | 32% | 28% |  |  |  |  |  |  |
|  |  | Tumor 3 | 61 | 50 | 111 |  | 0.078 | -0.078 | -0.444 | 0.289 |
|  |  |  | 6% | 6% |  |  |  |  |  |  |
|  |  | Tumor 4 | 45 | 22 | 67 |  |  |  |  |  |
|  |  |  | 4% | 3% |  |  |  |  |  |  |
|  | nodal_breast |  |  |  |  | <0.05 |  |  |  |  |
|  |  | Negative | 841 | 641 | 1482 |  |  |  |  |  |
|  |  |  | 70% | 78% |  |  |  |  |  |  |
|  |  | Positive | 357 | 183 | 540 |  | 0.219 | 0.219 | 0.105 | 0.332 |
|  |  |  | 30% | 22% |  |  |  |  |  |  |
|  | metastatic_breast |  |  |  |  | <0.05 |  |  |  |  |
|  |  | No | 1137 | 776 | 1913 |  | 0.282 | 0.282 | 0.121 | 0.443 |
|  |  |  | 92% | 88% |  |  |  |  |  |  |
|  |  | Yes | 94 | 107 | 201 |  |  |  |  |  |
|  |  |  | 8% | 12% |  |  |  |  |  |  |
|  | recurrent_progres_breast |  |  |  |  | <0.05 |  |  |  |  |
|  |  | Yes | 87 | 84 | 171 |  |  |  |  |  |
|  |  |  | 7% | 10% |  |  |  |  |  |  |
|  |  | No | 1199 | 775 | 1974 |  | 0.221 | 0.221 | 0.049 | 0.394 |
|  |  |  | 93% | 90% |  |  |  |  |  |  |
|  | stage_breast |  |  |  |  | <0.05 |  |  |  |  |
|  |  | Stage 0 | 163 | 81 | 244 |  | 0.254 | 0.254 | -0.038 | 0.546 |
|  |  |  | 13% | 9% |  |  |  |  |  |  |
|  |  | Stage I | 526 | 492 | 1018 |  | 0.235 | -0.235 | -0.527 | 0.057 |
|  |  |  | 43% | 53% |  |  |  |  |  |  |
|  |  | Stage II | 317 | 176 | 493 |  | 0.213 | 0.213 | -0.079 | 0.505 |
|  |  |  | 26% | 19% |  |  |  |  |  |  |
|  |  | Stage III | 125 | 70 | 195 |  | 0.176 | 0.176 | -0.116 | 0.468 |
|  |  |  | 10% | 8% |  |  |  |  |  |  |
|  |  | Stage IV | 101 | 104 | 205 |  |  |  |  |  |
|  |  |  | 8% | 11% |  |  |  |  |  |  |
|  | hormone_breast |  |  |  |  | <0.05 |  |  |  |  |
|  |  | ER/PR + HER2 + | 145 | 68 | 213 |  | 0.425 | 0.425 | 0.106 | 0.744 |
|  |  |  | 14% | 7% |  |  |  |  |  |  |
|  |  | ER/PR + HER2 - | 756 | 783 | 1539 |  | 0.208 | -0.208 | -0.527 | 0.111 |
|  |  |  | 71% | 78% |  |  |  |  |  |  |
|  |  | ER/PR - HER2 + | 59 | 35 | 94 |  | 0.266 | 0.266 | -0.053 | 0.585 |
|  |  |  | 6% | 3% |  |  |  |  |  |  |
|  |  | ER/PR - HER2 - (TNBC) | 110 | 120 | 230 |  |  |  |  |  |
|  |  |  | 10% | 12% |  |  |  |  |  |  |
| ***Lung*** |  |  |  |  |  |  |  |  |  |  |
|  | clinical_trial_available |  |  |  |  | <0.05 |  |  |  |  |
|  |  | Yes | 84 | 140 | 224 |  |  |  |  |  |
|  |  |  | 10% | 30% |  |  |  |  |  |  |
|  |  | No | 798 | 325 | 1123 |  | 0.777 | 0.777 | 0.612 | 0.942 |
|  |  |  | 90% | 70% |  |  |  |  |  |  |
|  | patient_status |  |  |  |  | <0.05 |  |  |  |  |
|  |  | New Patient | 805 | 423 | 1228 |  | 0.232 | 0.232 | 0.039 | 0.424 |
|  |  |  | 91% | 87% |  |  |  |  |  |  |
|  |  | Established Patient | 80 | 64 | 144 |  |  |  |  |  |
|  |  |  | 9% | 13% |  |  |  |  |  |  |
|  | histology_lung |  |  |  |  | 0.092 |  |  |  |  |
|  |  | Adenocarcinoma | 422 | 275 | 697 |  | 0.165 | -0.165 | -1.553 | 1.223 |
|  |  |  | 50% | 58% |  |  |  |  |  |  |
|  |  | Bronchoalveolar | 3 | 1 | 4 |  | 0.295 | 0.295 | -1.093 | 1.683 |
|  |  |  | 0% | 0% |  |  |  |  |  |  |
|  |  | Squamous cell carcinoma | 233 | 118 | 351 |  | 0.086 | 0.086 | -1.302 | 1.474 |
|  |  |  | 28% | 25% |  |  |  |  |  |  |
|  |  | Small cell carcinoma | 169 | 75 | 244 |  | 0.166 | 0.166 | -1.222 | 1.554 |
|  |  |  | 20% | 16% |  |  |  |  |  |  |
|  |  | Mesothelimoa | 9 | 6 | 15 |  |  |  |  |  |
|  |  |  | 1% | 1% |  |  |  |  |  |  |
|  | t_stage_lung |  |  |  |  | 0.234 |  |  |  |  |
|  |  | Tumor 1 | 219 | 69 | 288 |  | 0.166 | 0.166 | -0.129 | 0.462 |
|  |  |  | 27% | 22% |  |  |  |  |  |  |
|  |  | Tumor 2 | 271 | 113 | 384 |  | 0.045 | -0.045 | -0.340 | 0.251 |
|  |  |  | 34% | 36% |  |  |  |  |  |  |
|  |  | Tumor 3 | 160 | 74 | 234 |  | 0.110 | -0.110 | -0.406 | 0.185 |
|  |  |  | 20% | 23% |  |  |  |  |  |  |
|  |  | Tumor 4 | 149 | 60 | 209 |  |  |  |  |  |
|  |  |  | 19% | 19% |  |  |  |  |  |  |
|  | nodal_lung |  |  |  |  | 0.309 |  |  |  |  |
|  |  | Nodal 0 | 262 | 110 | 372 |  | 0.080 | -0.080 | -0.387 | 0.227 |
|  |  |  | 32% | 36% |  |  |  |  |  |  |
|  |  | Nodal 1 | 154 | 68 | 222 |  | 0.101 | -0.101 | -0.408 | 0.206 |
|  |  |  | 19% | 22% |  |  |  |  |  |  |
|  |  | Nodal 2 | 261 | 88 | 349 |  | 0.098 | 0.098 | -0.209 | 0.405 |
|  |  |  | 32% | 29% |  |  |  |  |  |  |
|  |  | Nodal 3 | 130 | 42 | 172 |  |  |  |  |  |
|  |  |  | 16% | 14% |  |  |  |  |  |  |
|  | metastatic_lung |  |  |  |  | <0.05 |  |  |  |  |
|  |  | No | 419 | 193 | 612 |  | 0.161 | 0.161 | 0.034 | 0.287 |
|  |  |  | 49% | 42% |  |  |  |  |  |  |
|  |  | Yes | 430 | 265 | 695 |  |  |  |  |  |
|  |  |  | 51% | 58% |  |  |  |  |  |  |
|  | recurrent_progres_lung |  |  |  |  | <0.05 |  |  |  |  |
|  |  | Yes | 49 | 64 | 113 |  |  |  |  |  |
|  |  |  | 6% | 14% |  |  |  |  |  |  |
|  |  | No | 813 | 379 | 1192 |  | 0.568 | 0.568 | 0.352 | 0.784 |
|  |  |  | 94% | 86% |  |  |  |  |  |  |
|  | genomic_status_lung |  |  |  |  | <0.05 |  |  |  |  |
|  |  | ALK + | 11 | 3 | 14 |  | 0.458 | 0.458 | -Inf | Inf |
|  |  |  | 5% | 2% |  |  |  |  |  |  |
|  |  | ALK - | 33 | 87 | 120 |  | 1.220 | -1.220 | -Inf | Inf |
|  |  |  | 14% | 59% |  |  |  |  |  |  |
|  |  | EGFR + | 19 | 15 | 34 |  | 0.156 | -0.156 | -Inf | Inf |
|  |  |  | 8% | 10% |  |  |  |  |  |  |
|  |  | EGFR - | 173 | 41 | 214 |  | 1.039 | 1.039 | -Inf | Inf |
|  |  |  | 72% | 28% |  |  |  |  |  |  |
|  |  | KRAS+ | 3 | 0 | 3 |  | Inf | Inf |  | Inf |
|  |  |  | 1% | 0% |  |  |  |  |  |  |
|  |  | KRAS- | 0 | 1 | 1 |  | Inf | -Inf | -Inf |  |
|  |  |  | 0% | 1% |  |  |  |  |  |  |
|  |  | CEA elevated | 2 | 0 | 2 |  |  |  |  |  |
|  |  |  | 1% | 0% |  |  |  |  |  |  |

ER/PR estrogen receptor/progesterone receptor; HER2: human epidermal growth factor receptor 2; TNBC: triple-negative breast cancer; ALK: anaplastic lymphoma kinase; EGFR: epidermal growth factor receptor; KRAS: KRAS biomarker; CEA: carcinoembryonic antigen
